# Response to “Phrase truncation in PubMed searches”

**DOI:** 10.5195/jmla.2017.261

**Published:** 2017-10-01

**Authors:** Steven Duffy, Shelley de Kock, Kate Misso, Caro Noake, Janine Ross, Lisa Stirk

**To the editor,** Shanman’s letter to the editor is absolutely correct to point out that phrases can be truncated when searching in PubMed. In our article, we wanted to depict, with a simplified search strategy, how it is not straightforward to translate a strategy from MEDLINE Ovid into PubMed syntax. It is important to note that the main focus of our article was not performance of truncation in PubMed; rather, we investigated whether supplementary searches of PubMed improved the currency of MEDLINE Ovid.

The actual MEDLINE Ovid search strategy used in practice had more synonyms and was designed to capture the terms “prostatic” as well as “prostate”:

(prostat$ adj3 (carcinoma$ or cancer$ or neoplas$ or tumo?r$ or malignan$ or adenocarcinoma$ or adenoma$)).ti,ab.

This was translated to run in PubMed as:

#1 “prostate cancer”[tiab] OR “prostate cancers”[tiab] OR “prostate cancerous”[tiab] OR “prostate carcinoma”[tiab] OR “prostate carcinomas”[tiab] OR “prostate neoplasm”[tiab] OR “prostate neoplasms”[tiab] OR “prostate neoplasia”[tiab] OR “prostate tumor”[tiab] OR “prostate tumors”[tiab] OR “prostate tumour”[tiab] OR “prostate tumours”[tiab] OR “prostate malignant”[tiab] OR “prostate malignancy”[tiab] OR “prostate malignancies”[tiab] OR “prostate adenocarcinoma”[tiab] OR “prostate adenocarcinomas”[tiab] OR “prostate adenoma”[tiab] OR “prostate adenomas”[tiab]
#2 “prostatic cancer”[tiab] OR “prostatic cancers”[tiab] OR “prostatic cancerous”[tiab] OR “prostatic carcinoma”[tiab] OR “prostatic carcinomas”[tiab] OR “prostatic neoplasm”[tiab] OR “prostatic neoplasms”[tiab] OR “prostatic neoplasia”[tiab] OR “prostatic tumor”[tiab] OR “prostatic tumors”[tiab] OR “prostatic tumour”[tiab] OR “prostatic tumours”[tiab] OR “prostatic malignant”[tiab] OR “prostatic malignancy”[tiab] OR “prostatic malignancies”[tiab] OR “prostatic adenocarcinoma”[tiab] OR “prostatic adenocarcinomas”[tiab] OR “prostatic adenoma”[tiab] OR “prostatic adenomas”[tiab]
#3 #1 or #2

With hindsight, we should have included prostat* in our concise example and described in detail how truncating more than one word in a phrase can have unexpected results in PubMed: neither prostat* cancer*[tiab] or “prostat* cancer*”[tiab] produce the expected results. Traditionally, we have used this method because of past experiences with unexpected results and issues with PubMed timing out. We have been overcautious with our use of quotation marks, as well as field tags, in order to disable PubMed’s Automatic Term Mapping so as to have more control over how our searches are performed.

Taking on board the approach that Shanman suggested, a modified version of our search strategy might look like the following:

prostate cancer*[tiab] OR prostate carcinoma*[tiab] OR prostate neoplas*[tiab] OR prostate tumor*[tiab] OR prostate tumour*[tiab] OR prostate malignan*[tiab] OR prostate adeno*[tiab] OR prostatic cancer*[tiab] OR prostatic carcinoma*[tiab] OR prostatic neoplas*[tiab] OR prostatic tumor*[tiab] OR prostatic tumour*[tiab] OR prostatic malignan*[tiab] OR prostatic adeno*[tiab]

Thank you for clarifying how phrase searching truncation is performed in PubMed. We will consider using this approach when designing search strategies for PubMed in the future.

Since our investigations were completed, Ovid has introduced the “Epub Ahead of Print” segment to their MEDLINE suite. This raises the question of whether it is still necessary to conduct supplementary searches in PubMed to identify ahead-of-print articles, which would be worth investigating further.

